# Evaluation of the setup accuracy of a stereotactic radiotherapy head immobilization mask system using kV on‐board imaging†

**DOI:** 10.1120/jacmp.v11i3.3192

**Published:** 2010-05-20

**Authors:** Imad Ali, Jesse Tubbs, Kerry Hibbitts, Ozer Algan, Spencer Thompson, Terence Herman, Salahuddin Ahmad

**Affiliations:** ^1^ Department of Radiation Oncology University of Oklahoma Health Sciences Center Oklahoma City OK 73104 USA

**Keywords:** stereotactic radiation therapy, thermoplastic immobilization mask, BrainLAB, on‐board imager, systematic and random errors

## Abstract

The purpose of this study was to evaluate setup accuracy and quantify random and systematic errors of the BrainLAB stereotactic immobilization mask and localization system using kV on‐board imaging. Nine patients were simulated and set up with the BrainLAB stereotactic head immobilization mask and localizer to be treated for brain lesions using single and hypofractions. Orthogonal pairs of projections were acquired using a kV on‐board imager mounted on a Varian Trilogy machine. The kV projections were then registered with digitally‐reconstructed radiographs (DRR) obtained from treatment planning. Shifts between the kV images and reference DRRs were calculated in the different directions: anterior‐posterior (A‐P), medial‐lateral (R‐L) and superior‐inferior (S‐I). If the shifts were larger than 2 mm in any direction, the patient was reset within the immobilization mask until satisfying setup accuracy based on image guidance has been achieved. Shifts as large as 4.5 mm, 5.0 mm, 8.0 mm in the A‐P, R‐L and S‐I directions, respectively, were measured from image registration of kV projections and DRRs. These shifts represent offsets between the treatment and simulation setup using immobilization mask. The mean offsets of 0.1 mm, 0.7 mm, and −1.6 mm represent systematic errors of the BrainLAB localizer in the A‐P, R‐L and S‐I directions, respectively. The mean of the radial shifts is about 1.7 mm. The standard deviations of the shifts were 2.2 mm, 2.0 mm, and 2.6 mm in A‐P, R‐L and S‐I directions, respectively, which represent random patient setup errors with the BrainLAB mask. The BrainLAB mask provides a noninvasive, practical and flexible immobilization system that keeps the patients in place during treatment. Relying on this system for patient setup might be associated with significant setup errors. Image guidance with the kV on‐board imager provides an independent verification technique to ensure accuracy of patient setup. Since the patient may relax or move during treatment, uncontrolled and undetected setup errors may be produced with patients that are not well‐immobilized. Therefore, the combination of stereotactic immobilization and image guidance achieves more controlled and accurate patient setup within 2 mm in A‐P, R‐L and S‐I directions.

PACS numbers: 87.56.‐v, 87.56.Da

## I. INTRODUCTION

Accurate tumor localization is essential for small intracranial or extracranial lesions that are treated with conformal^(^ ^1^ ^)^ or intensity‐modulated radiation therapy^(^ ^2^ ^)^ in order to achieve better tumor control and sparing of critical structures and normal tissue. Stereotactic radiotherapy with invasive patient immobilization is mostly used to treat small intracranial lesions with large single or hypofractionated dose.^(^ ^3^ ^,^ ^4^ ^)^ These patients are usually treated using precise, accurate and reproducible immobilization systems. Several commercial stereotactic systems developed by different vendors^(^ ^5^ ^–^ ^8^ ^)^ are available and commonly used to immobilize patients with intracranial lesions that are treated with stereotactic radiotherapy. A head ring^(^ ^9^ ^)^ is usually attached invasively with pins to the patient skull, which provides a reproducible patient setup between simulation and treatment with a positioning accuracy better than 2 mm. Other less invasive immobilization systems such as dental bite blocks, facial or head and neck masks^(^ ^7^ ^,^ ^10^ ^)^ are available for use to immobilize stereotactic radiotherapy patients. These systems are more flexible for treatments of lesions with broader dose regimen that include hypofractionated doses; however, the reproducibility and accuracy of patient setup is inferior to the rigid invasive head ring.^(^ ^3^ ^)^

Several studies have investigated the setup accuracy of these commercial stereotactic radiotherapy systems.^(^ ^7^ ^,^ ^9^ ^–^ ^11^ ^)^ With the development and clinical implementation of image‐guided radiation therapy techniques such as MV^(^ ^12^ ^,^ ^13^ ^)^ and kV on‐board imaging devices,^(^ ^14^ ^,^ ^15^ ^)^ new opportunities are available to perform rigorous evaluation of the accuracy of stereotactic radiotherapy immobilization and localization systems.^(^ ^16^ ^,^ ^17^ ^)^ The kV on‐board imaging system (OBI) provides diagnostic quality images with high position and contrast resolutions that can be employed to verify patient setup accuracy using patient's updated internal anatomy. In many clinics, the OBI is potentially used to obtain 2D projection or volumetric cone‐beam CT with patient current internal anatomy at the start of each treatment session. Then, these images are registered with reference 2D digitally‐reconstructed radiographs (DRRs) or volumetric CT obtained from treatment planning systems. The on‐line image registration tools allow anatomical matching between the two image sets and then three‐dimensional (3D) shifts between treatment and simulation setups are calculated. These shifts are then applied by moving the treatment couch to achieve anatomical match between reference and treatment images within a couple of millimeters of positioning accuracy.^(^ ^18^ ^)^

In this work we used kV on‐board imaging to evaluate patient setup and tumor localization accuracy of the BrainLAB stereotactic mask immobilization and localization system (BrianLAB, Inc, Westchester, IL 60154) used for intracranial lesions treated with a single or hypofractionated stereotactic radiotherapy. The patients were first set up using the BrainLAB immobilization mask and localizer and then kV imaging was performed independently. The shifts obtained by anatomical match using kV OBI image guidance were employed to quantify random and systematic errors of the stereotactic mask system.

## II. MATERIALS AND METHODS

### A.1 BrainLAB stereotactic mask and localizer

**Figure 1:**
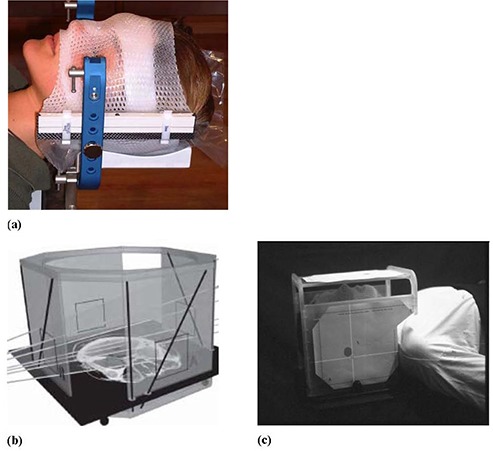
The stereotactic mask immobilization system (a); the BrainLAB localizer (b); target positioner (c) (from the BrainLAB hardware manual).

The BrainLAB mask system provides a noninvasive immobilization setup that allows fixation of patients treated with cranial stereotactic radiotherapy or radiosurgery. As shown in Fig. 1(a), the system consists of a head rest, a mask ring, vertical posts and a thermoplastic mask. The mask ring is attached to a couch‐mounted supporting system to provide rigid fixation of the patient. The vertical post and the head rest allow adjustable low or high fixation of the patient's head depending on the lesion location. The thermoplastic mask is composed from four immobilization components: (a) a lower layer that fixes the back of the head, (b) a middle layer with two strips, one under the nose and the second on the forehead, (c) a bridge that is shaped to take the patient's nose features, and (d) forehead and facial mask that is attached with the middle layer and nose‐bridge. The mask parts are snapped onto the vertical post with clips that have various thicknesses in the range from 1–4 mm. A middle thickness spacer (2 mm) is usually used in preparation of the mask and can be adjusted to smaller or larger spacer to compensate for mask loosening or shrinkage, respectively during the treatment course. The stereotactic head immobilization mask is shaped tightly to the head and used to treat brain lesions using single and hypofractions.

### A.2 Patient setup

Nine patients were simulated with the BrainLAB stereotactic head immobilization mask and CT localizer (Fig. 1(b)). The CT images were then moved to the treatment planning system (TPS). In the TPS, the isocenter coordinates were determined within the target using the BrainLAB localizer. Four overlays (A, B, C and D) are printed from the treatment planning that indicates the position of isocenter. These overlays were then attached to the target positioner that was mounted on the mask ring. The patients were set up using the BrainLAB target positioner (Fig. 1(c)) where the cross hairs on the overlays were aligned with the room lasers. The couch was moved initially to a position that lines up closely with isocenter and then it was locked. Final fine tuning of the couch position was performed using the micrometer adjustments of the couch‐mounted patient supporting system to align the crosshairs on the overlay printouts and room laser. Most of the patients represented here were treated for metastatic intracranial lesion using five hypofractions (5.0 Gy) to a total dose of 25.0 Gy. Nearly 90 patient setups using the BrainLAB mask and kV on‐board imaging were used in the data analysis.

### A.3 On‐board imager

**Figure 2:**
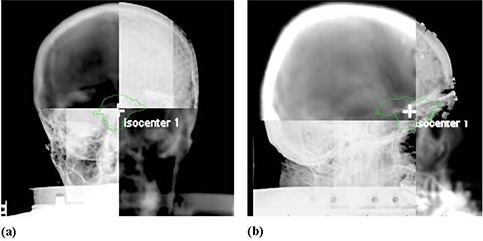
Image registration of orthogonal kV projections from the on‐board imager with DRRs from treatment planning system: (a) an anterior view; (b) a right lateral view.

The setup accuracy of the BrainLAB mask and localization system was evaluated using the kV on‐board imager (OBI) that is mounted on our Trilogy Linac (Varian Medical Systems, Palo Alto, CA). This imaging system consists of a diagnostic quality kV X‐ray source and an amorphous‐silicon flat‐panel imager (PaxScan 4030CB, Varian Medical Systems, Palo Alto, CA) that is mounted on the linac gantry and held by robotic arms. The imager has an effective area of $40\times 30\;{\text{cm}}^{2}$ and it was operated in 2×2 binning mode with resolution of 1024 × 786 pixels of the radiographic projections. The OBI was used to acquire an orthogonal pair of kV projection for the patients immobilized and localized using the BrainLAB stereotactic head mask and target positioner as shown in Figs. 2(a) and 2(b). The kV projections were then registered with a pair of DRRs obtained from the treatment planning system using the Varian Off‐Line‐Review software. Image registration was performed manually using bone markers that are within or close to the treatment area. The shifts were measured by one investigator (JT), and then a second investigator (IA) independently verified all shifts. We used side‐by‐side comparison, checker board, split screens image registration tools to measure the shifts. From our experience, automatic image registration tools did not work well in this investigation because of image artifacts from the metal frame of the stereotactic immobilization system. The shifts required to anatomically match the kV projections and DRRs were calculated. The treatment couch motion was locked after patient setup with BrainLAB system. If the shifts measured using the image guidance were larger than 2 mm, then the mask was taken off and the patient head was adjusted within the mask without moving the couch. A second orthogonal pair was acquired using the OBI and registered with DRRs, and then shifts between simulation and treatment isocenters were calculated to verify patient position. This process was repeated until the patient head setup in the mask was within 2 mm positioning accuracy based on patient internal anatomy obtained from OBI imaging. All shifts reported here are in the patient DICOM coordinate system with the positive lateral (x‐axis), vertical (y‐axis) and longitudinal (z‐axis), where the x‐axis increases to the left of the patient, y‐axis increases posteriorly, and z‐axis superiorly.

### A.4 Setup accuracy analysis

The shifts obtained from the kV image guidance were used to calculate the random and systematic errors associated with the BrainLAB immobilization mask and localizer. The statistical analysis approach employed in this work is similar to those reported in pervious works.^(^ ^7^ ^,^ ^13^ ^,^ ^19^ ^,^ ^20^ ^)^ The random errors were calculated from the standard deviation (σ) of the normal distributions that provide best fit to the shifts histograms in three dimensions: A‐P, R‐L and S‐I. These shifts were calculated by registering the kV radiographic images to DRRs acquired directly after patient setup and represent daily variation in patient setup. Systematic shifts in patient setup result mainly from different sources of errors that include: (a) systematic errors associated with each immobilization mask, (b) systematic errors of the BrainLAB localizer and target positioner, (c) OBI systematic error, and (d) alignment of room lasers with radiation isocenter. We have assumed that the systematic error of a mask depends on the mean of the normal distribution of the shifts for a particular patient in a certain direction. To extract the systematic errors of the mask, the mean was considered to result from simple addition of systematic errors from the different sources. For example, the mean of the normal distribution of the medial‐lateral shifts (along x‐axis) is given by the following:

(1) $$\Sigma_{x}^{\tau }=\Sigma_{1,x}+\Sigma_{2,x}+\Sigma_{3,x}+\Sigma_{4,x}$$

where $\Sigma_{1.x}$ is the mask systematic error, $\Sigma_{2,x}$ is the localizer systematic error, $\Sigma_{3,x}$ is the OBI systematic error, and $\Sigma_{4,x}$ is the systematic errors from the laser alignment with the radiation isocenter. The radial cumulative systematic error from the mask is obtained from quadratic addition of components in the different directions as follows:

(2) $$\Sigma_{1}={\left(\Sigma_{1,X}^{2}+\Sigma_{1,Y}^{2}+\Sigma_{1,Z}^{2}\right)}^{1/2}$$

The localization systematic error in a specific direction was assumed to depend on the mean of the normal distribution of the means of all masks for the different patients. This systematic error includes errors from BrainLAB CT localizer and target positioner, and it was called here, in short, as the localizer error. Analysis of the statistical significance of the means of the measured shifts in the different direction was performed using the t‐test of the null hypothesis at 95% confidence level.

The OBI systematic errors result mainly from sagging shifts of the gantry because of its weight.^(^ ^21^ ^)^ These errors were measured by a cubical phantom provided by the vendor (Varian Medical Systems, Palo Alto, CA) using standard OBI quality assurance procedures.^(^ ^22^ ^)^ This phantom is $5\times 5\times 5\times {\text{cm}}^{3}$ and has a spherical metal marker with a diameter of 2 mm placed at its center. The marker was aligned with radiation isocenter by aligning the room lasers with crosshair markers on the phantom surface. An orthogonal pair of kV projections (A‐P and R‐L views) was acquired of the cubical phantom. The 3D shifts of the metal marker were calculated by image registration with reference images that has the metal marker aligned to radiation isocenter. The measured shifts of 0.6 mm, −0.5 mm and 0.7 mm represent the OBI systematic error in A‐P, R‐L and S‐I directions, respectively. The room lasers were aligned with the radiation isocenter using the Winston and Lutz procedure.^(^ ^23^ ^)^ This includes alignment of a pointer phantom (provided by BrainLAB) with the radiation isocenter. Several radiographic projections for small fields ($1\;{\text{cm}}^{2}$) that are defined with the multileaf collimator are acquired at different gantry angles using the portal imager. The position of the pointer phantom is adjusted to be within 0.5 mm from the center of the field and this represents the accuracy of the alignment of the metal marker with the radiation isocenter. Then, the lasers are aligned with the crosshairs on the pointer phantom. The machining accuracy of the cubical phantom was tested by film radiographic imaging and it was found that the metal marker is located in the center of the phantom and aligned with the crosshairs on its surface within 0.1 mm. On our Trilogy machine, the room lasers reproduce the position of the radiation isocenter within 0.5 mm in the different directions. Systematic errors of the OBI were subtracted from the means in order to obtain net systematic errors for the mask and localizer according to Eq. (1); however, the small errors from the laser alignment were ignored.

## III. RESULTS

**Figure 3:**
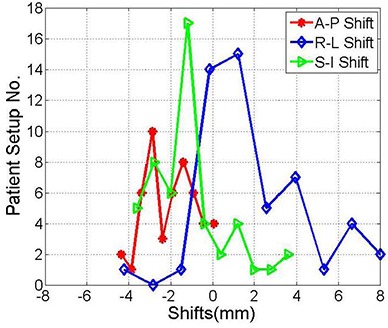
Histograms of the shifts between the kV‐images acquired on the treatment machine and the DRRs from the treatment planning system in the anterior‐posterior, right‐left, superior‐inferior directions, respectively. These shifts were obtained from image registration directly after patient setup with the BrainLAB mask immobilization and localization system.

Figure 3 shows histograms of the shifts from nearly 50 patient setups using the BrainLAB immobilization mask and localization system obtained from kV OBI image guidance. Shifts as large as 4.5 mm, 5.0 mm, 8.0 mm in the A‐P, R‐L and S‐I directions, respectively, were measured from image registration of kV projections and DRRs. These shifts result from different sources that include random and systematic errors. The standard deviations of 1.2 mm, 2.5 mm, 1.8 mm represent random errors of patient setup in the A‐P, R‐L and S‐I directions, respectively, using the BrainLAB mask. The means of −2.0 mm, 2.0 mm, and −1.2 mm in the A‐P, R‐L and S‐I directions, respectively, include cumulative systematic errors of the OBI, BrainLAB mask and localizer as shown in Table 1. The shift distributions in the different directions are significantly different from a normal distribution, with a mean of zero based on the p‐values of the null hypothesis at 95% confidence level, as shown in Table 1.

**Table 1:** Mean and standard deviation the anterior‐posterior, right‐left, and superior‐inferior shifts from the histograms shown in Fig. 3.

	*A‐P (mm)*	*R‐L (mm)*	*S‐I (mm)*
Mean	−2.0	2.0	−1.2
Stand. Dev.	1.2	2.5	1.8
P‐Value	0.00001	0.00001	0.00006

**Figure 4:**
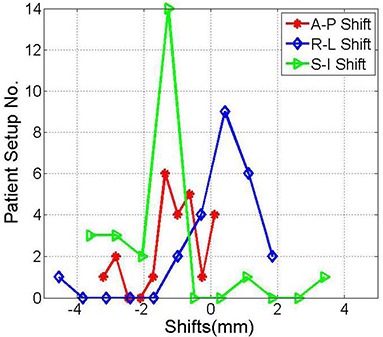
Histograms of the shifts obtained from image registration of kV projections and the DRRs in the anterior‐posterior, right‐left and superior‐inferior directions, as indicated. These shifts are the same as in Fig. 1 with the exclusion of one individual patient with frequent setups.

One patient was treated with normal fractionated regimen using the BrainLAB immobilization mask and localizer for 26 treatment sessions. This patient represented a special case where she was not setting up straight because of a tilt in her neck. Therefore, including this patient in the above data shown in Fig. 3 caused a statistical bias in the calculated means and standard deviations of the shift distributions. The histograms of the shifts in Fig. 4 represent the shifts measured by image guidance for the same patients as in Fig. 3 excluding the setups that belong to this individual patient. As illustrated in Table 2, means and standard deviations of the shift are smaller than when considering all patients as in Table 1. The shifts in the different directions are significantly different from zero based on the p‐values of the null hypothesis at 95% confidence level, except in the R‐L direction (p‐value=0.26350).

**Table 2:** Mean and standard deviation of the anterior‐posterior, right‐left, and superior‐inferior shifts for the distributions shown in Fig. 4.

	*A‐P (mm)*	*R‐L (mm)*	*S‐I (mm)*
Mean	−1.1	0.3	−1.6
Stand. Dev.	0.9	1.3	1.6
P‐Value	0.00001	0.26350	0.00010

Table 3 shows the systematic errors for eight BrainLAB immobilization masks and the localizer used to set up stereotactic radiotherapy patients. Seven patients were treated with five fractions and one patient was treated with a single fraction. The mean was calculated from nearly five data points for the hypofractionated treatments. However, for the patient with a single fraction, the mask systematic error was extracted from a single point measurement. The BrianLAB localizer systematic error was calculated from the mean of the normal distribution of the mean shifts of each individual patient setup as shown in the last row in the different directions. The systematic errors of the OBI and laser alignment were extracted from the means of the shifts measured for all treatments per patient. The resultant was considered a measure of the systematic error of the masks in the different directions as shown in rows 1–9 (Table 3).

**Table 3:** Systematic errors of nine BrainLAB immobilization masks and localizer in the different directions.

*Mask*	*A‐P (mm)*	*R‐L (mm)*	*S‐I (mm)*	*Radial (mm)*
1	−2.3	3.1	−0.3	3.9
2	−1.6	−0.4	−1.9	2.5
3	−1.0	0.4	3.3	4.4
4	−1.1	0.6	−0.8	1.5
5	−0.6	0.0	−0.7	0.9
6	−0.3	0.3	−0.5	2.9
7	0.1	0.3	−0.5	0.6
8	−0.3	−0.2	−2.0	2.0
9	−2.8	−0.6	−3.2	4.3
Localizer	−1.1	0.4	0.9	1.4

**Figure 5:**
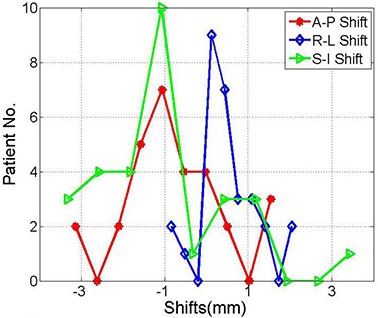
Histograms show the shifts between the kV projections acquired on the treatment machine and the DRRs from the treatment planning system in the anterior‐posterior, right‐left and superior‐inferior directions, respectively. The kV projections were obtained after patient setup modification using kV image guidance.

Figure 5 represents the shifts obtained from image anatomical matching using kV OBI imaging after modification of patient position in the immobilization mask. The standard deviation of the normal distributions measured were 1.2 mm, 0.7 mm, 1.7 mm and 1.0 in the A‐P, R‐L, S‐I and radial directions, respectively. The means were −0.7 mm, 0.5 mm, −1.0 mm and 2.5 mm in the A‐P, R‐L, S‐I and radial directions, respectively, as shown in Table 4. This shows that with image guidance using kV OBI imaging, both random and systematic errors that are measured by standard deviations and means, respectively, have been improved in nearly all directions. This is mainly due to correction of the random errors and mask systematic errors by resetting the patient in the immobilization mask based on image guidance. However, systematic errors of the OBI and image registration program were not removed and still cause inaccuracy in patient setup.

**Table 4:** Mean and standard deviation of the anterior‐posterior, right‐left, and superior‐inferior shifts after patient position correction within the immobilization mask guided by kV on‐board imaging shown in Fig. 5.

	*A‐P (mm)*	*R‐L (mm)*	*S‐I (mm)*
Mean	−0.7	0.5	−1.0
Stand. Dev.	1.2	0.7	1.6
P‐Value	0.00320	0.00150	0.00260

**Figure 6:**
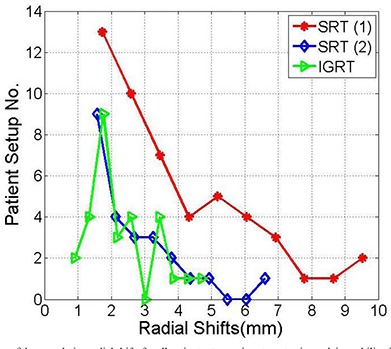
Histograms of the cumulative radial shifts for all patient setups using stereotactic mask immobilization and localization (SRT (1)), patient stereotactic setup excluding one patient with many setups (SRT (2)), and combining stereotactic setup and image guidance (IGRT) as indicated.

Figure 6 shows that the mean and standard deviation of the cumulative radial shifts are smaller by excluding the patient with large systematic error. The mean and standard deviations of cumulative radial shift are reduced further using image guidance in combination with the BrainLAB mask immobilization and localization. The outlier points in the tails are significantly smaller combining image guidance with stereotactic patient setup. In all cases, the means of the radial shifts were significant considering the null hypothesis of the mean at 95% confidence level as shown in Table 5.

**Table 5:** Mean, standard deviation and p‐value of the cumulative radial shifts of the histograms shown in Fig. 6.

	*Radial Shifts SRS (1)*	*Radial Shifts SRS (2)*	*Radial Shifts IGRT*
Mean	3.9	2.4	2.3
Stand. Dev.	2.2	1.3	1.0
P‐Value	≤0.00001	≤0.00001	≤0.00001

## IV. DISCUSSION

Patient setup accuracy with the BrainLAB immobilization mask and localization system is limited by different sources of errors that include random and systematic errors. The statistical analysis represented in this work provides a method to separate and quantify the contribution of random and systematic errors from different sources such as BrainLAB immobilization mask and localizer, OBI, and laser alignment with radiation isocenter. Random daily errors result from personnel judgment in setting up the patients with stereotactic immobilization mask and localization system. These errors can be reduced by establishing setup protocols, and by better training and education of the medical staff on the use of this system. The largest errors that we have measured were systematic errors that resulted from the masks used for patient immobilization according to the shifts calculated using kV imaging as shown in Table 3. These errors highlight the importance of the preparation process of the mask before CT simulation and patient setup on the treatment machine. Tight masks should be molded to achieve accurate tumor localization and reasonable patient comfort. The stereotactic localization system that include CT localizer and target positioner should be checked during acceptance testing and related systematic errors should be minimized. Other sources of errors are the OBI, laser alignment and treatment couch motion. The OBI systematic errors can not be detected by image guidance procedure and require independent tests to quantify the associated errors (as explained above in Material and Methods A.3). The systematic errors from laser alignment with radiation were measured using the Winston‐Lutz system and found to be within 0.5 mm, and their contribution to the cumulative error was smaller than OBI, mask and localizer errors. It is important to correct the major sources of error in order to achieve more accurate patient setup.

In contrast with the head ring which employs invasive patient immobilization and is appropriate only for single fraction treatment, the mask is a noninvasive immobilization system that provides the clinic with a more flexible tool to treat stereotactic patients with single or multiple fractions. However, based on the imaging data from the kV on‐board imager, significant shifts were measured sometimes when the patients were set up using only the stereotactic immobilization mask and localization system. Previous studies^(^ ^7^ ^,^ ^10^ ^)^ reported large shifts using the BrainLAB mask immobilization, and showed that combining dental mold or upper jaw support with the mask improved the accuracy of patient setup in agreement with this work. Other studies measured the shifts required using the Exac‐Track system for patients who were immobilized using the BrainLAB immobilization mask system^(^ ^7^ ^)^ and reported large shifts to correct patient position.

In this work, we would like to stress the importance of image‐guided radiation therapy using kV on‐board imaging for patients immobilized and localized with stereotactic systems such as the BrainLAB mask. Even with more rigid immobilization systems such as the head ring,^(^ ^9^ ^)^ dental molds, or upper jaw supporting system,^(^ ^7^ ^,^ ^10^ ^)^ the setup accuracy may be compromised by uncontrolled offsets that result from ring slippage. An independent verification system of the patient setup is important where secondary shifts might be detected and corrected. Image‐guided radiation therapy with the kV on‐board imager provides shifts that are required to set up the patient at the time when the images were acquired prior or during patient treatment. Application of these shifts will correct both random and systematic errors from the stereotactic immobilization and localization systems. This leads us to propose that both techniques of patient stereotactic immobilization and localization combined with setup using kV on‐board image guidance are required for patient setup. The combination of rigid immobilization and image guidance before and during the treatment session will achieve more controlled and accurate patient setup, where patients are well‐immobilized and the treatment is delivered to the spot based on what is seen by kV imaging.

The setup accuracy that we were able to achieve with image guidance was limited by locking the couch motion which was employed following the tradition of stereotactic radiation therapy. The couch was not moved to apply the shifts calculated by image registration of the kV orthogonal pair and DRRs. In our approach, we changed patient position within the mask in order to obtain better anatomical match based on kV image guidance. Image guidance was used only to verify that the shifts calculated by on‐line image registration in each direction was less than 2 mm. Patient setup accuracy may be improved by unlocking the couch and correcting the anatomical shifts that are calculated by on‐line image registration. However, this will require evaluation of the couch position reproducibility as well as accuracy and ability to perform kV image verification of patient setup, because most of the intracranial stereotactic patients are treated with different couch positions. In this work, 2D projections were acquired using the kV on‐board imager and registered with reference 2D images from DRRs created in TPS. 2D image guidance is limited by poor visibility of the soft tissue structures and inferior contrast resolution. Further, with 2D image registration, only three translation shifts are used to set up the patients. 3D imaging using on‐board kV cone‐beam CT and comparison with CT images from treatment planning provides better visibility of soft tissue and bones. Further, it is easier to determine 3D rotational offsets on 3D CBCT than 2D projections. Thus, further investigation is required to improve the outcome of patient setup using stereotactic immobilization and localization combined with image guidance using 3D imaging.

## V. CONCLUSIONS

The BrainLAB mask provides a noninvasive, practical and flexible immobilization system to treat single and hypofractionated doses for intracranial stereotactic radiosurgery or therapy. Relying on this system for patient setup only might be associated with significant positioning errors. On the other hand, image guidance with the kV on‐board imager provides shifts that are required to set up the patient at the time when the images were acquired prior to or during patient treatment. The patient may relax or move during treatment, and thus uncontrolled and undetected setup errors may be produced with patients who are not well‐immobilized. The combination of stereotactic immobilization and image guidance achieves more controlled and accurate patient setup.
